# eHealth, Participatory Medicine, and Ethical Care: A Focus Group Study of Patients’ and Health Care Providers’ Use of Health-Related Internet Information

**DOI:** 10.2196/jmir.3792

**Published:** 2015-06-22

**Authors:** Anne Townsend, Jenny Leese, Paul Adam, Michael McDonald, Linda C Li, Sheila Kerr, Catherine L Backman

**Affiliations:** ^1^ Arthritis Research Canada Richmond, BC Canada; ^2^ Department of Occupational Science and Occupational Therapy University of British Columbia Vancouver, BC Canada; ^3^ Mary Pack Arthritis Program Vancouver Coastal Health Vancouver, BC Canada; ^4^ Maurice Young Centre for Applied Ethics School of Population and Public Health University of British Columbia Vancouver, BC Canada; ^5^ Department of Physical Therapy University of British Columbia Vancouver, BC Canada; ^6^ Arthritis Patient Advisory Board Arthritis Research Canada Richmond, BC Canada

**Keywords:** relational ethics, health-related Internet information, arthritis, patient-HCP relationship, e-health, chronic illness, qualitative

## Abstract

**Background:**

The rapid explosion in online digital health resources is seen as transformational, accelerating the shift from traditionally passive patients to patients as partners and altering the patient–health care professional (HCP) relationship. Patients with chronic conditions are increasingly engaged, enabled, and empowered to be partners in their care and encouraged to take responsibility for managing their conditions with HCP support.

**Objective:**

In this paper, we focus on patients’ and HCPs’ use of health-related Internet information and how it influences the patient-HCP relationship. In particular, we examine the challenges emerging in medical encounters as roles and relationships shift and apply a conceptual framework of relational ethics to examine explicit and nuanced ethical dimensions emerging in patient-HCP interactions as both parties make increased use of health-related Internet information.

**Methods:**

We purposively sampled patients and HCPs in British Columbia, Canada, to participate in focus groups. To be eligible, patients self-reported a diagnosis of arthritis and at least one other chronic health condition; HCPs reported a caseload with >25% of patients with arthritis and multimorbidity. We used a semistructured, but flexible, discussion guide. All discussions were audiotaped and transcribed verbatim. Elements of grounded theory guided our constant comparison thematic analytic approach. Analysis was iterative. A relational ethics conceptual lens was applied to the data.

**Results:**

We recruited 32 participants (18 patients, 14 HCPs). They attended seven focus groups: four with patients and three with rehabilitation professionals and physicians. Predominant themes to emerge were how use of health-related Internet information fostered (1) changing roles, (2) patient-HCP partnerships, and (3) tensions and burdens for patients and HCPs.

**Conclusions:**

Relational aspects such as mutual trust, uncertainty, and vulnerability are illuminated in patient-HCP interactions around health-related Internet information and the negotiated space of clinical encounters. New roles and associated responsibilities have key ethical dimensions that make clear the changes are fundamental and important to understand in ethical care. When faced with tensions and burdens around incorporating health-related Internet information as a resource in clinical encounters, participants described a particular ambivalence illustrating the fundamental changes being negotiated by both patients and HCPs.

## Introduction

eHealth technology is widespread and wide-ranging [1] and is transforming health care delivery [2-4]. According to the World Health Organization, “eHealth is the cost-effective and secure use of information and communications technologies in support of health and health-related fields, including health-care services, health surveillance, health literature, and health education, knowledge and research” [5]. eHealth includes an array of resources and devices [6-12], with the capacity to provide patients with extensive and up-to-date information, access to medical research [13], connections to people with similar conditions, immediacy, and convenience in patient-health care professional (HCP) communications [13,14] and improved health outcomes [6,15]. The proliferation of eHealth strategies is accelerating a shift in health care from a traditional and paternalistic delivery model to a more mutual patient-HCP relationship, where informed patients are actively involved in their care and treatment decisions [16,17]. This shift supports a patient-centered model of care based on effective communication, respect for patients and shared patient-HCP responsibility in care provision and management of long-term conditions [18].

While many patients use health-related Internet information (HRII), the information may or may not be shared in medical consultations. The Pew Research Center’s Internet & American Life Project reported that 35% of US adults reported going online specifically to diagnose a condition for themselves or someone they knew, and 72% of Internet users reported they looked online for health information within the past year. Over half (53%) of online diagnosers said they talked with a medical professional about what they found online [19]. While advancing patient empowerment and shared decision-making has been identified as a practical and moral necessity [20], uncertainty exists about how HRII is affecting the cornerstone of good medical care, that is, the patient-HCP relationship [21]. As Anderson et al noted over a decade ago, “While the use of the Internet does hold considerable promise for health care, it raises a host of social and ethical issues that need to be addressed. In particular, professional resistance to the use of this new technology in health care remains one of the greatest barriers to realization of the Internet’s ample potential” [22]. There is growing evidence about how HRII is used in consultations [23-25]. For a range of reasons, however despite patients’ sourcing HRII, it may not feature in consultation discussions [26]. When HRII is discussed in medical consultations, it can have both positive and negative impacts [27] and does not always mark a shift toward patient-centeredness [28], particularly as patient and HCP perspectives on the role of HRII can differ [29]. While patients may be motivated to spend time and effort seeking HRII about specific diseases and treatments, physicians face constraints searching relevant information for individual patients [30] and HCP responses to use varies [31]. While HRII has the capacity to advance a partnership model of care [32], it is not clear how changing roles and responsibilities for both patients and HCPs [33] are negotiated in these shifting times.

HRII has the potential to empower patients to adopt a heightened sense of individual autonomous action [34,35], and this has fundamental implications for patient-HCP relationships in both explicit and nuanced ways. The interpersonal requirements of achieving constructive and respectful partnerships [36] may be underestimated and subsequently not well examined or easily understood. As Agarwal et al noted, patient-HCP interactions in consultation discussions involve the “critical interplay” of traditional values such as trust and responsibility, and a reliance on an oversimplified model often neglects new patient-HCP dynamics and the impact of these changes on patient-HCP relationships [37].

A relational ethics lens provides a conceptual framework to better understand the relational shifts in ethical patient-HCP relationships. Relational ethics [38] builds on traditional bioethical principles of autonomy, justice, beneficence, non-maleficence [39], and shifts attention to relationships as the source of ethical action [40]. As greater emphasis is placed on how patients and HCPs negotiate communications and shared decision-making, a relational lens [41,42] provides contextual and relationship insights into health care practices [43]; this is particularly pertinent in chronic illness care [44] and patient-centered care [32,45]. Core elements of relational ethics are mutuality, engagement, respect, trust, vulnerability, uncertainty, and an interdependent environment [40] applicable to everyday experiences, practices, and interactions. Our current knowledge on the ways the relational shift (changing relationships) is happening for both patients and HCPs is limited. We need to better understand the meaning, process, and context of the clinical encounter for those involved and the potential risks and benefits of HRII to advance effective patient-HCP relationships.

In this paper, we present findings from the focus group phase of our study, which was designed to examine the influence of different types of eHealth use on patient-HCP relationships. We limit analysis here to the discussions on HRII. We make explicit the ethical dimensions underpinning the dynamics of evolving patient-HCP relationships as the use of HRII grows.

## Methods

### Research Design

This focus group qualitative study [1] was designed to explore relational aspects of eHealth for patients and HCPs. Our aim was to better understand how participants perceived and experienced different types of eHealth. A major focus to emerge in the discussions was how changes were occurring in patient-HCP relationships arising from use of HRII in the context of chronic illness care. Ethical relationships are integral to health care, and so a conceptual framework of relational ethics suited our aims and objectives.

### Context

**Figure 1:**
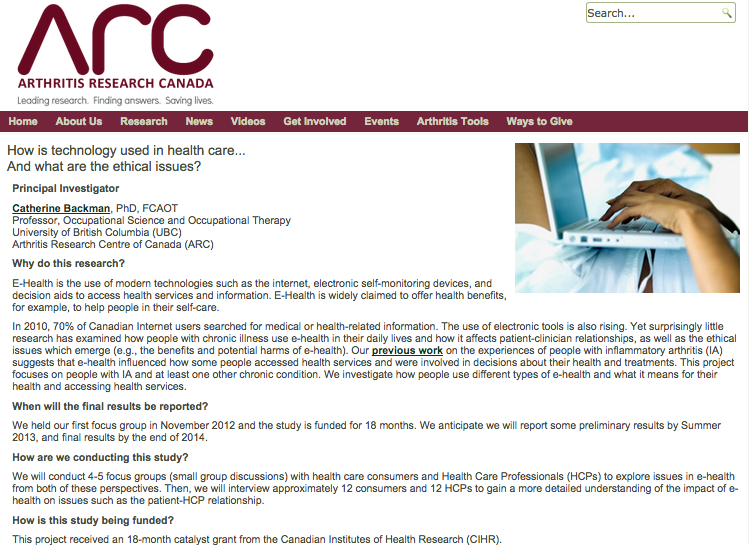
Online recruitment document.

Being based at an arthritis research center influenced recruitment and feasibility of the study (Figure 1). Participants were recruited in two urban areas of British Columbia, Canada, between November 2012 and June 2013 for focus group discussions via online arthritis sites, for example, the Arthritis Research Center (ARC), the Arthritis Patient Advisory Board (APAB), Web and social media sites (Twitter and Facebook), posters in clinical settings, and word-of-mouth. All sessions were held in the greater Vancouver area except for one HCP group that was held in Victoria. Focus groups were held in community centers, health care centers or participants’ places of work. The University of British Columbia’s Behavioral Research Ethics Board granted approval. Participants gave written consent.

### Sampling and Recruitment

The 32 participants consisted of 18 adult patients (16 female, 2 male) and 14 HCPs (11 female, 3 male) who participated in seven focus groups. Four groups were held with 4-6 patient participants, aged in their 30s-70s. Three groups were held with 4-5 HCP participants: physical and occupational therapists, a rheumatology nurse, a laboratory technician and rheumatology fellows, aged in their 30s-60s. Patients were recruited based on a self-reported diagnosis of arthritis, plus at least one other chronic condition. The majority of participants were middle class and Caucasian, which does not fully represent the diversity of the geographic region. The HCPs included physicians and rehabilitation providers who cared for people with multiple chronic conditions. We selected people with arthritis and co-conditions for this study for two reasons: pragmatism (being based in an arthritis research center) and prevalence. Arthritis is a highly prevalent and severe chronic condition globally and the leading cause of pain and disability in Canada [46]. The Canadian Community Health Survey (CCHS) (124,844 respondents, response rate 76%), based on 2007-2008 data, estimated that more than 4.2 million Canadians 15 years and older (16% of the population) had arthritis [46]. The coexistence of other chronic conditions with arthritis was reported as common by the Public Health Agency of Canada, based on the CCHS 2007-2008 data. Multimorbidity is associated with high burdens of care and cost [47]. Despite this, our knowledge and understanding of the impact of multimorbidity for patients and HCPs is poor [48]. Because eHealth (including HRII) is a vast resource for both patients and HCPs, it is vital to identify its potential benefits and harms, perhaps particularly salient for those who have multimorbidity and their HCPs who manage extensive information and encounter increasingly complex decisions [1]. A more detailed rationale is given in the published protocol [1]. All participants completed a demographic form that included data on ownership and use of digital devices. All participants used tablets, desk computers, or laptops for HRII.

### Procedure

In the focus groups, eHealth was defined broadly as any digital health technologies that people used or had any knowledge of. Participants were asked to identify the range of devices and reasons they used them at the start of each discussion. This was identified on flip chart papers and guided the discussions. Ethics was framed as pros and cons of eHealth with a particular focus on patient-HCP relationships and was elaborated on depending on how the discussions unfolded. This was to avoid fixed definitions of ethics constraining the discussions. The topic guide was devised to explore patient and HCP experiences of eHealth use in chronic illness and was arranged around four key areas: (1) What types of eHealth and devices do you use? (2) What sort of things do you use eHealth for? (3) How does eHealth influence what you do regarding your health and illness conditions (patients) or practice (HCPs), including your interactions with HCPs/patients? and (4) What do you see as the benefits and drawbacks of eHealth?

We encouraged group discussion [28] where participants voiced their priorities and concerns. We attempted to tease out eHealth use in the practical circumstances of living with chronic illness or chronic illness care. We introduced relational ethics aspects by probing the nature of patient-HCP interactions and relationships, shared decision-making, and self-managing. Discussions were audiotaped, facilitated by AT or PA (authors), and lasted approximately 2 hours. Focus groups were transcribed verbatim, checked against recordings for accuracy, and de-identified.

### Data Analysis

An iterative, thematic approach using constant comparative methods was applied to the data. All data were coded, with no preset categories. AT and JL (authors) independently read and annotated transcripts, identifying emerging issues for discussion as data collection proceeded. We modified and added codes in light of fresh transcripts and repeated readings. When all focus groups had been completed, PA and CB (authors) read a selection of the transcripts and shared their observations based on the original aims of the study, the topic guide, and shared interpretations as analytical discussions continued among research team members. In this way, the analysis was both inductive and deductive. We applied initial broad themes to all transcripts, and these were outlined to all research team members (including researchers, practitioners, and patients) who asked further questions and offered additional interpretations. Early common themes emerged across the patient and HCP data such as changing roles and responsibilities; collaboration, notions of trust; and teamwork. After further analysis, we agreed on higher-level themes, for example, partnerships. We then organized them into predominant themes: changing roles, with subthemes of “being prepared” and “responsibilities”; “partnerships”, which had a subtheme of “trust”; and “tensions and burdens”.

In planning the study, we agreed on conceptualizations of relational ethics as an overarching analytic conceptual framework as applicable to our research question. As in all qualitative studies, there are multiple ways to analyze and interpret data. In this primary analysis of focus group findings, the application of relational ethics helped us focus on what is valued in interactions and relationships and what is at risk, rather than specific aspects of eHealth like the nature of self-monitoring devices. Relational ethics informed the analysis in relation to how participants discussed patient-HCP roles and relationships and prized values, for example, roles and responsibilities, partnerships, mutual trust, and vulnerabilities. In extracts shared here, patient groups are notated PG, health care professionals groups are notated HCP, and within each group participants are given a number (P1 to P6).

## Results

### Overview

This analysis is limited to the discussions around HRII and how this influenced patient-HCP roles and relationships. The types of HRII reported by patients were Internet searches in general, health-related websites (eg, universities, health organizations, non-profit disease-oriented organizations), aimed at both patients and HCPs. To a lesser extent, patients also reported using personal websites and blogs (eg, individuals sharing personal experiences and resources), chat rooms (eg, open and closed groups on specific sites or via social media platforms like Facebook), and online links to medical test results (eg, to an eHealth record at the lab that did their blood-work). HCPs reported a similar range of Internet resources but described less frequent and extensive searches, and their resources were largely limited to health- and professional-oriented websites. HCPs did not cite use of blogs and chats as frequently as did patients. They reported a tendency to rely on a few specific online resources pertinent to their practice, some of which they recommended to patients. A few HCPs used online sites for sharing medical tests results. For both patients and HCPs, the factual content of the HRII they used was largely about research and treatment options, medication self-management strategies, and resources. Patients also sought information about diagnosis and sought the experiences of others with similar conditions/symptoms.

Both patients and HCPs relayed how HRII prompted interactive and negotiated communications. A traditional paternalistic era of care was contrasted with a partnership model illustrating patient involvement in discussions, but also tensions around change at the level of patient-HCP relationship. Here we present three predominant themes, the first two with subthemes that offer additional depth with specific examples: (1) changing roles, with subthemes of “being prepared” and “responsibilities”, (2) partnerships, with a subtheme of trust, and (3) tensions and burdens. Although for organizational purposes we identify three themes, they are overlapping and interlocking, for example, the changing roles and new responsibilities contribute to a partnership model of care, which in turn is characterized by particular tensions and burdens. We then discuss the ethical issues that emerge as patient-HCPs relationships evolve.

### Changing Roles

#### Overview

Both patients and HCPs identified information from hospital or university sites as reliable sources of HRII. However, they also accessed other HRII and described the information as extensive, provisional, uncertain, and contradictory. Patient participants discussed being more informed but more uncertain, which prompted more questions about their health conditions and treatment options. They recognized that HCPs could not know everything, and they no longer conceived of them as the gatekeepers of knowledge. Whereas “in the old days, the doctor was God…[today] doctors don’t know that much at all…I don’t think any of us in the world realized that before…Before we would accept what the doctor said but in the last ten years, we’ve had access to the information and now we question more” (P3, PG3). Other patient focus groups concurred with the challenge to health professionals of increasing amounts of information and informed patients, given the limited time for clinical visits. This had implications for their view of the patient role. They did not rely on their HCP as their information source. Rather, in the context of ongoing illness, repeated visits to different HCPs, and increasingly expert in their conditions, they expressed the patient role as being equipped with information and enabled to ask questions and take part in an interactive dialogue about care and treatment. To participate fully, they had to be prepared for the medical encounter.

#### Being Prepared

Both patients and HCPs discussed the key role of HRII in preparing patients for medical consultations. They discussed potential practical and ethical benefits, for example, how more informed patients saved time and were enabled to be actively involved in discussions about treatment decisions. Reflecting experiences across the illness trajectory, patients described how they prepared for consultations with their general practitioner (GP) and specialists or allied health professionals for specific reasons (eg, to gain a referral or to get advice on treatments or exercise). Using HRII, they felt newly equipped to interact in focused and effective ways. This made “a huge difference…finding information, and what it means, before you go to the doctor so you can have an intelligent conversation…[and] ask them the right questions” (P3, PG3). Another patient compared how her consultation style had changed: “Before it was…‘I ache’”, whereas with the benefit of HRII, “It’s allowed me to…narrow it down…‘I want that, that and that clarified’…and I won’t ask or talk about other things” (P2, PG2). This participant described “working” on her next visit about “what medications and how to ask for them and how to report back which ones are not working” (P2, PG2). These words indicate the need for patients to spend time and effort and develop skills in order to be fully involved in the decision making process.

Being prepared also had explicitly relational benefits for patients: “I talk to them [GP and rheumatologist] about what I feel…and what I found [online]…And it’s a very respectful relationship with both of those doctors to me…the rheumatologist he is now listening to me a little more because he knows that I’m doing my homework” (P5, PG1). This is another indication of changing roles and relationships—being prepared for the consultation for this patient meant gaining respect from the HCP.

HCPs agreed that prepared patients allowed a more interactive consultation in which patients were equipped to ask relevant questions about care and treatment. One HCP described “a stellar client” who uses HRII and then emails her questions to follow-up in the consultation which “kind of gets me prepared, she’s prepared” (P3, HCPG2). Another HCP reflected the positive aspects of HRII for both patient and HCP roles: “Much of it is empowering patients via education. So them coming and having done their pre-reading…gives you more time to focus on what you’re going to assess, treat, and actually impact” (P1, HCPG2). HCPs noted patient preparation as key to more effective and time efficient consultations, bringing focus, and allowing the HCP more time to provide good care.

#### Responsibilities

Patient participants discussed how being prepared for consultations made a difference to the care they received. For some, this meant an added responsibility for their patient role. One participant noted how:

“Bringing [HRII] stuff in…makes a difference in the treatment because…it seems like I just get the basics unless I’m doing the pro-activeness…So it’s just my responsibility to look into what I think might work and then ask for their opinion” (P6, PG3).

Searching for and sharing information in the consultation was framed as not only helping themselves, but their doctor, and in some cases other patients: “[Doctors are] not miracle makers…They don’t think about certain things…We do have a responsibility to come to them with that [information] because not only are we helping ourselves, we’re helping them to help other people” (P1, PG2).

Being responsible information-seeking patients had its risks and meant assessing the reliability of HRII for oneself. One patient participant spontaneously framed this as an ethical responsibility:

The ethics are…with yourself because you make the final decision…based on information that you get from all different sources…You…check the…authenticity…You try to find references to the same product or theory…By the end you have to decide for yourself. The government can’t protect you from it. I don’t know who else could except yourself.

P3, PG1

This extract illustrates one example of how some patients stated the importance of patients (and not only HCPs) to act ethically in new ways. It is clear from this patient’s perspective that it is the patient’s responsibility to gather the information they need to deal with health concerns and to be equipped with information in order to participate in focused discussion in the medical encounter. This implies a shift in the burdens of responsibility from the HCP to the patient. From a relational ethics perspective, it is questionable as to how reasonable and fair such a shift is for all patients. But what we note here is a perception of redistributed responsibilities. Not all patients in our study were wholly comfortable with this level of responsibility.

### Partnerships

Patients, all of whom had multiple health conditions, discussed teamwork and emphasized the importance of effective communications between themselves and multiple HCPs. One patient expressed the mechanisms underlying partnering with a team of HCPs:

P3, P5 and P4, you touched on something very important…when we’re diagnosed, we do the research even for our other un-arthritis related ailments…You’re the captain of your team you take that [HRII] back to your doctor or your rheumatologist…and they know your total package. And then [you] encourage the physio, the occupational [therapists]…your pharmacist and your other specialist, your dentist…It all interacts…all parcel of…having an inflammatory disease. But they all have to be on the same page…it’s your body, it’s your stuff happening…you’re the one that kind of brings them all together…to make sure that…there is that inter-professional [collaboration] going on.

P2, PG1

One patient conveyed how HRII equipped her to change the power balance, be heard, and get support from HCPs, noting “It’s ammunition” (P1, PG2). Being heard, however, could be reliant on the relationship and how the HCP responds:

It depends…how good your doctor is…if your doctor understands that you have Internet access and that you have some understanding of what you are reading then it’s a mutual respective relationship…But if your doctor doesn’t understand…you probably need to look for another doctor…It’s really not something you want to do when you have multiple chronic conditions. But…sometimes it’s necessary because you’ve got to find somebody that will understand that you have this history and you understand a certain amount and you’re wanting to work towards controlling and managing it. And you need his support.

P3, PG3

Reflecting the patient views, the HCP groups also discussed changes in the patient-HCP relationship. In some cases, patients brought HRII into the consultation and it prompted discussion, which in turn fostered a partnership form of interaction:

You just have to be really open to the fact that they’re [patients] going to tell you things you didn’t know and that’s great. “Oh I hadn’t seen that before. That might be useful for me with other clients”. So I definitely feel it’s more of a partnership…[like] P2 says it’s much less didactic…Like P5 said, you just put in context what they’ve already brought to the table.

P1, HCPG2

Another HCP group discussed how “It’s not the old medical hierarchy…(Now) we’re negotiating...‘What have you read? You’ve seen that, cool, let me see it.’ So it’s way more interactive because of the abundance of knowledge” (P2, HCPG1). It was clear that some consultations were changing to a more mutual information exchange and interactive discussion, based on HRII introduced by the patient or websites recommended by HCPs to patients: “Some of those websites have been successful in complementing and supplementing my care and helping to improve the partnership aspect of care” (P5, HCPG2).

In the context of partnership building, there was some evidence to suggest the need to negotiate a balance between ethical concerns of autonomy and beneficence. The HCPs discussed how respecting informed patients’ decisions could mean avoiding “pushing” what they as HCPs saw as more medically effective (see quote below). There was a suggestion that patients are more equipped and enabled to take part in discussions. For HCPs this meant negotiating a new space, providing medical and health advice while respecting potentially differing opinions. This indicates the need for HCPs to develop new skills in consultations. One HCP described some of the consequences of informed patients, in the context of HRII and the balance between a more traditional approach and a more patient-led approach:

All we can do is inform people, give them our best medical opinion. And they have to make their own decision based on what they’ve heard from us and what other information they have. So I don’t think you’re always successful in convincing them to your side but I think we try pretty hard not to push our opinions on too.

P2, HCPG3

Participants described a negotiated element of interactions. One HCP described how new roles involved looking at information patients brought to the consultation, weighing up its relevance to the planned session and how credible the information sounds, while being respectful of the patient contribution: “It’s trying to find a common path to work forward” (P3, HCPG2). Another HCP noted:

By giving her [patient] that list of websites and saying, “Okay this will be a partnership. I…will look for the things that might be triggering this [pain]. But I want you to look for and learn about the things that might be amplifying it. And if we agree to do this, this will be our partnership…” The websites…did enhance and improve the partnership aspect. I’m learning from her and she’s learning from me…That’s one of the ways of how it’s affected my relationship.

P5, HCPG2

#### Trust

For patients, trust was key to sharing HRII with HCPs in consultations and encouraged them to share: “Because you trust them [HCPs], you’re taking it [HRII] to them…” (P2, PG1). This sharing included learning to interact in a more open way than previously, for example, sharing concerns about medication use: “I’ve learned to be a bit more open with him [rheumatologist]…honest…Describing the side effects or my fears about a drug and…not continuing with it” (P2, PG2). Patients also appreciated HCPs being open about what they did not know as this built trust: “I think it’s hard for some of them to say, ‘I don’t know that. I’ll need to refer you.’ Which I think is the smartest answer. That makes me have a lot of faith in a doctor” (P2, PG2).

HCPs also described how HRII could build trust:

If you spend that last five minutes…showing them [patients]…“This is a website that you can read too. It’s got enough information but not too much and it won’t overwhelm you. This is endorsed by the Canadian Arthritis Society.” It kind of builds a level of trust and…adds a component of enrichment to the appointment… they read about it and I think they just feel a lot more like, empowered and cared for … equipped.

P1, HCPG3

In this sense, trust was based on a partnership model of care whereby the patient trusts the HCP to support her becoming informed, equipped, and empowered via guided direction to and interpretation of HRII.

### Tensions and Burdens

Although the patient discussions around HRII were mostly positive, there were downsides: “It may be a burden to find all this information if you don’t have the time, the energy, or the physicality” (P1, PG1). This situation raised tensions for patients: on the one hand, they saw HRII as an important resource, but many also noted negative aspects. One patient described online searching as “frustrating” and “a struggle” and stopped searching commenting: “I believe in the ostrich approach” (P4, PG4). For this patient with multiple conditions, the search for HRII became too burdensome to continue. Although patients expressed feeling informed, in control, and empowered, their HCP was a key resource to guide, verify, and advise and “have that funnel” (P2, PG1) to clarify their thinking. In this way, the role of the HCP was as an expert guide who could ease the navigation struggles through an array of HRII. Patients also noted that discussions required the HCP to “be ready” to engage with them. One patient felt that family doctors found it “threatening…if we look things up…GPs have to accept this as the new patient…We’re going to…find out as much as we can…And then we’re going to go in and ask the questions. And they better be ready” (P3, PG3).

HCPs agreed it was key to support patients in their Internet use, but there were associated tensions, for example, negotiating time constraints: “I have to find ways to give them [patients] as much knowledge in as short amount of time…the amount of information that’s appropriate and not excessive” (P2, HCPG2). This also meant being sensitive to the needs of the patient with multiple chronic conditions and symptoms such as pain, fatigue, and “brain fog”, or there was a risk of patients “falling off the system”. HCPs acknowledged that patients might bring information to consultations that could be a little “out of their realm”. Several HCPs described how important it was to welcome this development: “I’ve…decided that right upfront if somebody has clearly done way more reading into an area that I’d ever done I just say: ‘Wow, you know more about that than I do’…It’s really important not to feel threatened by that information because…if you [did]…that will affect your relationship” (P2, HCPG2).

## Discussion

### Principal Findings

Our focus group findings indicate practical and ethical implications of using HRII in chronic illness care, as care needs and patient-HCP relationships evolve. Patients and HCP discussed their changing roles and responsibilities [3] and expressed how HRII equips patients to engage in discussions with HCPs, who reflected on new ways of listening and responding to patients in light of HRII accessed by both parties. In practical terms, this mutual engagement required that patients work to prepare for consultations advancing a partnership model of care [23,49,50]. Our findings also highlight the importance of trust in the effective use of HRII in collaborative patient-HCP relationships. For example, the relational shift from a traditional hierarchical relationship to a more reciprocal relationship was associated with burdens and tensions. The presence of multimorbidity for patients involves multiple medications, risks, appointments, self-management strategies, and HCPs. These cascading complexities together with rapidly evolving HRII and increased expectations placed on patients, infuse shared decision-making. How patients and HCPs relate to one another as roles and responsibilities evolve requires careful consideration. Hence, understanding the detail of the relational aspects of medical encounters are key to ethical and effective care. Both patients and HCPs conveyed medical encounters as “negotiated spaces” where mutual vulnerabilities were revealed as patients and HCPs sought to relate to each other in a changing context of care. In this context, mutual trust could foil tensions.

Our findings resonate with previous studies of how eHealth technologies impact patient-HCP roles and relationships in a range of ways. HRII can support the decision-making process [51], yet there remains potential to provoke tensions [27,52,53] and HCPs can experience anxiety around sharing HRII with patients [54]. One literature review about the impact of HRII and patient-HCP relationships [55] concluded that a shift in the role of the patient from passive recipient to active consumer of health information prompted three types of HCP response: they felt threatened and were defensive, they collaborated in accessing the information, or they guided patients to reliable health information websites. The HCP participants in our study tended to agree on the importance of collaboration and guidance to foster care, yet acknowledged it was difficult to find the time to do so efficiently.

Another focus group study of patients with chronic illness [56] reported that patients saw the Internet as an additional resource to support valued relationships with their doctors and conveyed no desire to disrupt the existing balance of power in the consultation. We found something different. Our findings indicate patient empowerment as present and valued by patients, and that for some patients and HCPs, the balance of power in medical encounters shifted with the use of HRII. Valued relationships involved not just support and guidance from HCPs, but recognized mutual vulnerabilities. For example, patient trust in HCPs was strengthened when HCPs were open about not knowing, and patients felt confident in sharing with HCPs the HRII they had found. This finding differs from research that reported trust could be hampered due to increased patient knowledge and empowerment [57]. For the patient participants in our study, HRII prompted greater interdependence between patients and HCPs and increased opportunities for partnerships, which in turn were underpinned by key ethical values of mutual trust and respect—cornerstones of shared decision-making. This supports findings from a recent focus group study of patients with complex chronic conditions in Canada that identified the need for open two-way dialogue to improve coordination of “whole person” care [18], which may be particularly important for those with multiple conditions with holistic needs [58].

Our findings also showed how patients and HCPs learned techniques to negotiate an evolving consulting space and avoid conflict. For HCPs, this includes a dimension of risk and uncertainty about how to offer appropriate guidance or how to acknowledge that the patient may know more than them. This reflects Zufferey et al’s [28] perspective indicating a need to work at engagement by increasing professionals’ level of reflexivity—a continuous and demanding “work” to adjust one’s behavior to others. We also found that patients did work—the homework or preparation work that interactive medical encounters required resonates with previous sociological research on the unending work of chronic illness [59] including moral dimensions [60] and ethical perspectives on the high degree of patient work needed for collaborative decision-making [61]. Our findings suggest new aspects of unending work for both patients and HCPs as they navigate extensive HRII sources and negotiate new ways of interacting with each other.

Being “overloaded” with “endless” HRII has been documented elsewhere [62,63] and resonates with our findings. Taking a relational approach, we emphasize the implications of this for the roles and responsibilities of HCPs who are now required to guide those patients who may feel overwhelmed, and without relevant support may choose to take the “ostrich approach” and withdraw from HRII seeking. Our findings, however, also suggest that in some cases, patients’ use of HRII can point to open, collaborative patient-HCP relationships; some HCPs in our focus groups described how they recommend and discuss HRII with patients as part of practice. This mirrors aspects of recent research [63], which concludes that patients need skill development [63] in order to use HRII effectively. A relational ethics approach recognizes the burdens and vulnerabilities of HCPs too [40], and acknowledges that HCPs also require guidance, training, and skill development if they are to use HRII effectively.

Values that underpin health care are fundamentally ethical in nature. Core relational values identified in this study of mutuality, responsibility, honesty, and trust provide the foundation for ethically appropriate and effective care. These values were consistent across the patients and HCPs. A relational lens encourages consideration of the details of patient-HCP dynamics and reveals how interactions can support or obstruct the realization of prized values that are emerging in HRII consultations [37]. In presenting an ethical analysis, our paper contributes to the sparse literature that explicitly addresses ethical challenges of medicine and health on the Internet [64], in terms of patient-HCP relationships. This perspective also aligns with Medlock et al, who see patient empowerment as a “practical and moral necessity” [65].

### Limitations and Strengths

Although we aimed to recruit a diverse sample of participants in order to examine a range of experiences of using HRII, our sample was not as varied as intended. Proportionately, we lacked views from male and younger patients, and transferability of findings is limited accordingly. We were able to compare, however, HCP and patient views and experiences of HRII and gain an understanding of the key emerging ethical and relational aspects specific to living with and caring for people with the complexity of multiple health conditions. The findings presented here are also limited to use of HRII and are not implications of other eHealth tools and devices.

### Practice Implications

The relational ethics framework applied in this study may help advance evolving patient and HCP partnership roles consistent with the value of patient-centered care. Drawing on mutual respect and a more reciprocal understanding of relationships in terms of sharing knowledge, providing support, and recognizing vulnerabilities [40] should inform reflective practice and health care communications. Relational ethics then offers guidance to HCPs in their everyday clinical practice (thinking, reasoning, and decision-making) and expands the professional ethics and responsibility that already guide practice [32]. The promotion of shared decision-making is a key component of ethical patient-centered care [41] and is also regarded as a way to optimize the use of scarce resources in health care [32].

Given the complexity of managing information for multiple health conditions from multiple sources, patients reported varying levels of comfort with responsibility for shared decisions. While HCPs are generally aware of patient vulnerabilities, our findings nevertheless point out the need for negotiating this shared decision-making space—a considerable challenge in many practice contexts given time constraints and busy practices. This is highlighted when comparing more active, engaged patients with the patients who are less so in terms of self-management and shared decision-making. HCPs need to be aware of how some people with multiple chronic conditions will be “captain of their ship”, take ownership/control, and perhaps be more vigorous in shared decision-making than others. This places a burden on the HCP to understand their patients’ preferences and styles and to be aware of a range of HRII that patients may find helpful in order to engage in shared decision-making. Examining relational positioning is key to understanding changing patient-HCP relationships. Our findings offer a better understanding of the patient-HCP relationship and communications in the context of the rapid adoption of HRII.

### Suggestions for Research

Research, guidelines, and theories about the adoption of HRII have not kept pace with technological developments, adoption of eHealth tools and devices, and increasing ease of access to information [13]. This presents a pressing need for a better understanding of the complexities emerging in the evolution of role and relationships of patients and HCPs including complex and everyday clinical decision-making. Relational understandings emphasize mutual vulnerability and interdependence needed for effective teamwork and encourage detailed consideration of health care interactions and patient autonomy in patient-HCP relationships and how for example HCPs can support and undermine patients’ autonomy [42]. In their response to patients’ preparation for visits, HCPs, perhaps unknowingly, have the potential to undermine a patient’s identity and self-evaluation (which are basic to their sense of being able to function autonomously). They have the opportunity, however, to encourage patients to seek and use autonomy-supporting resources such as relevant websites or patient groups [42]. Our findings revealed that patients used the HRII to equip them with information to participate meaningfully in discussions in medical consultations. This interactive process suggested increasingly interdependent and reciprocal relationships between patients and HCPs.

### Conclusion

The impact of HRII on the patient-HCP relationship will continue to grow. Considering future implications, our research offers new insights into how both patients and HCPs adopt strategies to better negotiate the changing nature of the patient-HCP relationship. Our findings add a new dimension to shared decision-making in the era of HRII by emphasizing the fundamental relational and ethical aspects, including the negotiations, vulnerabilities, and trust that are part of an ongoing process in clinical encounters for a partnership model. Relational ethics is a novel approach that in this context attends to the moral space where this care occurs.

## Abbreviations

GP: general practitioner
HCP: health care professionals
HRII: health-related Internet information
